# Event Representations and Predictive Processing: The Role of the Midline Default Network Core

**DOI:** 10.1111/tops.12450

**Published:** 2019-09-04

**Authors:** David Stawarczyk, Matthew A. Bezdek, Jeffrey M. Zacks

**Affiliations:** ^1^ Department of Psychological & Brain Sciences Washington University; ^2^ Department of Psychology, Psychology and Neuroscience of Cognition Research Unit University of Liège

**Keywords:** fMRI, Default network, Self‐generated thoughts, Mind‐wandering, Event cognition, Predictions, Naturalistic stimuli, Attention

## Abstract

The human brain is tightly coupled to the world through its sensory‐motor systems—but it also spends a lot of its metabolism talking to itself. One important function of this intrinsic activity is the establishment and updating of *event models*—representations of the current situation that can predictively guide perception, learning, and action control. Here, we propose that event models largely depend on the default network (DN) midline core that includes the posterior cingulate and anterior medial prefrontal cortex. An increasing body of data indeed suggests that this subnetwork can facilitate stimuli processing during both naturalistic event comprehension and cognitive tasks in which mental representations of prior situations, trials, and task rules can predictively guide attention and performance. This midline core involvement in supporting predictions through event models can make sense of an otherwise complex and conflicting pattern of results regarding the possible cognitive functions subserved by the DN.

## Abstract 2

**Stawarczyk, Bezdek, and Zacks** offer neuroscience evidence for a midline default network core, which appears to coordinate internal, top‐down mentation with externally‐triggered, bottom‐up attention in a push‐pull relationship. The network may enable the flexible pursuance of thoughts tuned into or detached from the current environment.

## 1 Introduction

How does the brain place itself in a position to deal with the twists and turns of complex situations? One possibility is that it builds generative models of current situations and then uses these representations to drive predictions about what will happen in the near future. Building and updating such representations is an expensive proposition, but it can pay off in the form of more effective anticipatory responses, better learning, and improved ability to use previous experiences to guide current understanding and action (Richmond & Zacks, 2017).

In this paper, we will make a speculative proposal about how the brain operates to build models of ongoing situations, which is based in large measure on new results from functional magnetic resonance imaging (fMRI) studies measuring resting state functional connectivity (Fox & Raichle, 2007), stimuli‐locked correlations (Hasson, Nir, Levy, Fuhrmann, & Malach, 2004), and multivariate pattern analyses (Norman, Polyn, Detre, & Haxby, 2006). We will ultimately propose that the midline default network (DN) core actively and continuously builds models of the immediate situation that enable prediction and interpretation. To do so, we will first detail two apparently conflicting views of the possible cognitive functions subserved by the DN (Sections 2 and 3), and then describe how the current proposal can reconcile them (Section 4). We will also consider the relationship of this proposal to related theoretical efforts (Section 5), and how functions may be differentiated *within* the midline DN core (Section 6) before summarizing our main claims in a brief conclusion section (Section 7).

## 2 Posterior midline DN areas as a locus for self‐generated thoughts and internal attention

**Figure 1:**
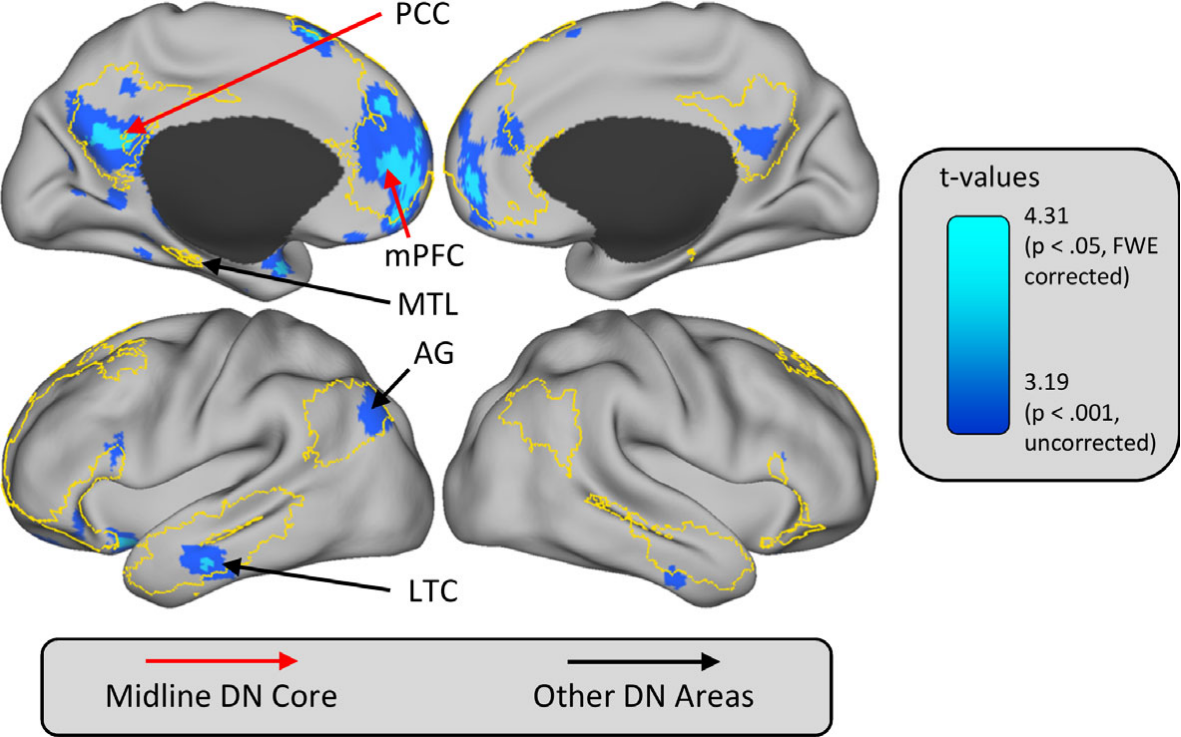
Illustration of the DN as defined by resting‐state functional connectivity and task‐based contrast analyses. The yellow border illustrates the DN as defined by the seven‐network resting‐state parcellation of Yeo et al. (2011). The red arrows indicate the midline DN core areas; the black arrows indicate DN areas that are not part of the midline core. The overlaid activations show brain regions that are more activated in the contrast of self‐reports of mind‐wandering versus being fully focused on‐task, from Stawarczyk, Majerus, Maquet, et al. (2011). The dark blue activations are thresholded at *p* < .001, uncorrected for multiple comparisons. The teal activations are thresholded at *p* < .05 family‐wise error (FWE) corrected for multiple comparisons over the DN mask. PCC, posterior cingulate cortex; mPFC, medial prefrontal cortex; MTL, medial temporal lobe; AG, angular gyrus; LTC, lateral temporal cortex. Activations are displayed on an inflated surface map (population average landmark surface: PALS‐B12) using CARET software (Van Essen, 2005).

The DN is a set of brain regions that include the posterior cingulate cortex (PCC), the medial prefrontal cortex (mPFC), the angular gyrus, the lateral temporal cortex, and areas in the medial temporal lobes, (see Fig. 1). The main features of this network are, first, that its level of activation as measured with positron emission tomography (PET) or fMRI generally decreases during cognitive tasks that involve focused attention to external stimuli compared to the resting state (Gusnard & Raichle, 2001; Shulman et al., 1997) and, second, that the activity of its different constituents fluctuates synchronously during rest (Greicius, Krasnow, Reiss, & Menon, 2003). Together, these features suggest that the DN may support cognitive processes that are suspended during task performance. However, given the unconstrained nature of cognitive activity in the absence of an actual task, there has been a continuous debate about the possible cognitive functions underpinned by this network (e.g., Spreng, 2012). Functions proposed to be attributed to the DN have included the monitoring of interoceptive and exteroceptive perceptions (Gusnard & Raichle, 2001), self‐referential processing (Gusnard, Akbudak, Shulman, & Raichle, 2001), conceptual semantic processing (Binder et al., 1999), and the free association of mental content retrieved from memory to form a “stream of thought” (Andreasen et al., 1995).

**Figure 2:**
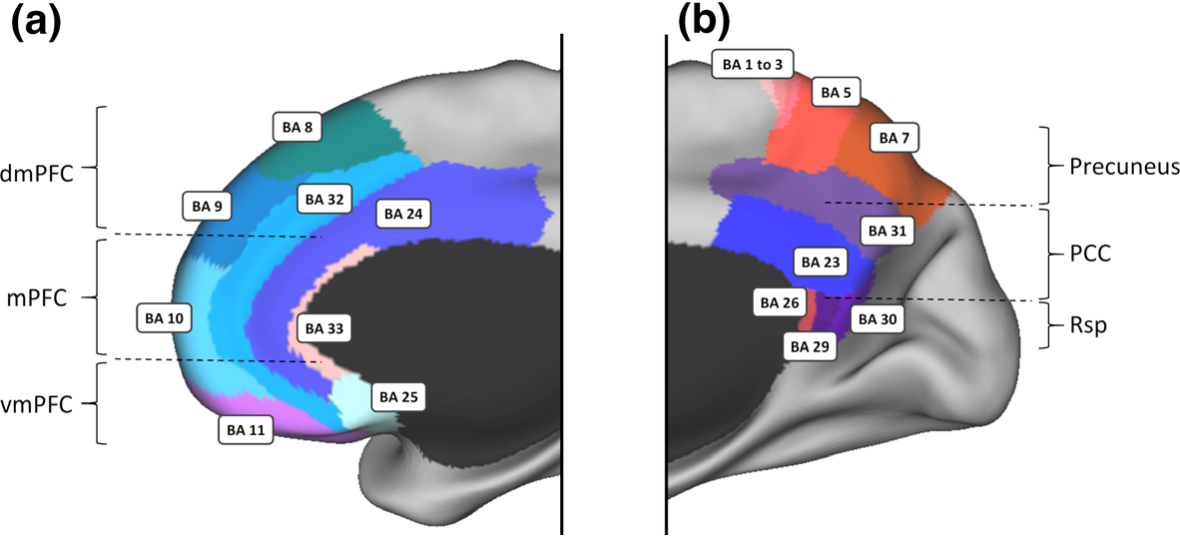
Anatomy of the midline DN core based on Brodmann cytoarchitecture. Panel (a) illustrates the approximate location of the mPFC, which is often described as primarily comprising areas 32, 24, and 25 (Córcoles‐Parada et al., 2017). However, recent parcellations of the DN consider that the mPFC can be subdivided into three parts (dmPFC, mPFC, and vmPFC) with only the mPFC being part of the DN midline core. The dmPFC is more strongly associated with regions involved in mentalizing and social cognition, while the vmPFC is more closely related to limbic and subcortical areas supporting motivational and valuation processes (Andrews‐Hanna et al., 2010; D’Argembeau, 2013; Yeo et al., 2011). Areas 12 and 14 are not represented as they were not explicitly defined in humans by Brodmann (1909). Panel (b) illustrates the approximate location of the PCC, which was initially considered as consisting of areas 23, 29, 30, and 31 (Brodmann, 1909). However, more recent views consider that the PCC only includes the posterior parts of areas 23 and 31 (Leech & Sharp, 2014; Vogt, 2009) with areas 29 and 30 being parts of the Rsp. Area 7 is usually referred to as the Precuneus, while areas 1, 2, 3, and 5 are parts of the sensory‐motor system. dmPFC, dorsal medial prefrontal cortex; mPFC, medial prefrontal cortex; vmPFC, ventral medial prefrontal cortex; PCC, posterior cingulate cortex; Rsp, retrosplenial cortex. The Brodmann areas are displayed on the PALS‐B21 inflated surface map using CARET software (Van Essen, 2005).

FMRI studies of task activations and functional connectivity parcellate the DN into several subcomponents (Andrews‐Hanna, Reidler, Sepulcre, Poulin, & Buckner, 2010; Yeo et al., 2011). Among these, the functions of the midline core areas, particularly the PCC, have remained the most elusive. The PCC has one of the highest basal energy consumption levels of the brain (Gusnard & Raichle, 2001; Leech & Sharp, 2014) and is characterized by a dynamic pattern of functional connectivity with a wide array of cortical areas. The PCC is highly connected to other regions within the DN, forming a midline core subsystem with the mPFC where both regions act as major cortical hubs that integrate information across the network (Andrews‐Hanna et al., 2010; Buckner et al., 2009; see Fig. 2 for an anatomical delineation of these two regions). In addition, recent studies have revealed that the PCC also shows transient periods of cross‐network interactions with a number of other brain regions (Kabbara, Falou, Khalil, Wendling, & Hassan, 2017; de Pasquale, Della Penna, Sporns, Romani, & Corbetta, 2016). This has led to the proposal that the PCC not only constitutes a major regional hub within the DN but also behaves as a connector hub that integrates information originating from most of the brain (de Pasquale, Corbetta, Betti, & Della Penna, 2018; Leech, Braga, & Sharp, 2012; van den Heuvel & Sporns, 2013). These features likely explain why the PCC is associated with a variety of cognitive tasks in the spatial, motivational, social, and memory domains (e.g., Bzdok et al., 2015; Leech & Sharp, 2014; Pearson, Heilbronner, Barack, Hayden, & Platt, 2011).

Regarding the possible functions of the midline DN core, one dominant view is that, through their interaction with other DN areas, the PCC and mPFC mainly support self‐generated thoughts that are decoupled from the here and now (Andrews‐Hanna, Smallwood, & Spreng, 2014; Buckner & Carroll, 2007; Christoff, Irving, Fox, Spreng, & Andrews‐Hanna, 2016; Smallwood et al., 2016). Given the limited nature of attentional resources, it has been proposed that the suppression of self‐generated thoughts and internal mentation is required for optimal performance during demanding tasks that require the continuous processing of external stimuli (Smallwood & Schooler, 2006). During such tasks, higher activity in the PCC has been associated with impaired performance (Weissman, Roberts, Visscher, & Woldorff, 2006), and activity in the midline DN core has been associated with self‐reports of mind‐wandering episodes (Christoff, Gordon, Smallwood, Schooler, & Smith, 2009; Stawarczyk, Majerus, Maquet, & D’Argembeau, 2011; see Fig. 1). In addition, fMRI studies using functional connectivity analyses have shown that activity in the DN at rest is usually anticorrelated with activity in regions involved in processing external stimuli (Fox et al., 2005; but see Dixon et al., 2017); this has led to the proposal of a push–pull relationship between internal mentation and external attention (Kelly, Uddin, Biswal, Castellanos, & Milham, 2008; Sestieri, Shulman, & Corbetta, 2010, 2017). Finally, recent studies have assessed brain areas on a gradient ranging from unimodal regions involved in sensory/motor processing to transmodal regions involved in more abstract processing and showed that the midline DN areas are at the top of this hierarchy (Margulies et al., 2016; Sepulcre, Sabuncu, Yeo, Liu, & Johnson, 2012). These findings suggest that these regions are particularly well‐suited to processing transmodal information unrelated to immediate sensory input. Such a claim is widely supported by meta‐analyses of fMRI studies that show large overlaps in midline DN activations across a range of tasks requiring internally focused mentation based on long‐term memory representations; these include autobiographical memory, self‐projection in the future, episodic memory retrieval, mind‐wandering, mentalizing, semantic cognition, and personal goal processing (Benoit & Schacter, 2015; Binder et al., 1999; Kim, 2012, 2016; Spreng, Mar, & Kim, 2009; Stawarczyk & D’Argembeau, 2015).

In sum, task‐based and functional connectivity fMRI studies have led researchers to the view that the midline DN core specializes in self‐generated, offline representations and stands in a push‐pull relationship with other systems that focus on processing the current external world (Chun, Golomb, & Turk‐Browne, 2011; Dixon, Fox, & Christoff, 2014; Smallwood, Brown, Baird, & Schooler, 2012; Sonuga‐Barke & Castellanos, 2007).

## 3 The midline DN can guide the processing of external stimuli by maintaining previously constructed representations

The conceptualization of the DN as a system that focuses inward and trades off control with systems focused on the outside world may be the dominant one in current theorizing—but it is not the only view. An alternative proposal holds that the midline DN can play crucial roles in the processing of external stimuli by combining new information with previously constructed representations. Evidence for this view comes from studies in which stimulus processing depends upon integrating current visual information with information in memory. For example, in a variant of the n‐back task that did not require memory updating (0‐back condition), better performance was associated with reduced activity in the midline DN core (Smallwood et al., 2013). This finding would appear to support the push–pull view. However, when participants had to continuously update their mental representations of the stimuli and maintain them in mind to correctly answer the targets (1‐back condition), better performance was associated with *increased* DN activity (Murphy et al., 2019; see also Konishi, McLaren, Engen, & Smallwood, 2015; Spreng et al., 2014). Complementary results come from a study that showed higher performance when degraded pictures were preceded by their original versions in an identification task (González‐García, Flounders, Chang, Baria, & He, 2018). Importantly, compared to degraded pictures that were not preceded by the originals, this effect of prior knowledge on performance was reflected at the neural level by (a) higher activity levels in the midline DN core and (b) activity patterns in these regions that were both more distinct from each other and more similar to those elicited by the original pictures.

In addition, midline DN core activity and connectivity have also been found to be greater when ongoing performance is guided by a recently acquired rule. In one recent study (Vatansever, Menon, & Stamatakis, 2017), participants performed a switching task in which they were instructed to sort cards based on an undisclosed dimension (color, shape, number, or identity) that they had to discover by trials and errors after each switch. Activity in the midline DN core was higher following the discovery of the currently relevant dimension than in the preceding trial‐and‐error period. Furthermore, greater functional connectivity between a seed placed in the PCC and primary visual cortex, hippocampus/parahippocampal gyrus, and amygdala was associated with faster response times (RTs) but only after the new relevant dimension had been found out. This suggests that the midline DN core was involved in applying newly learned rules to guide performance (for other studies involving the PCC and mPFC in maintaining and applying learned rules in switching and working memory tasks, see Crittenden, Mitchell, & Duncan, 2015; Koshino, Minamoto, Yaoi, Osaka, & Osaka, 2014; Smith, Mitchell, & Duncan, 2018).

In sum, in contrast to the view that the midline DN areas exclusively support self‐generated mentation that stands in a push–pull relationship to the processing of external stimuli, a body of data suggests the midline DN core might also be involved in maintaining mental representations of task rules and previously presented stimuli to guide behavior and facilitate task performance (for a related proposal, see also Margulies & Smallwood, 2017). This suggests that these regions play a key role in integrating internally generated representations—including information and knowledge retrieved from long‐term memory—with recently acquired information from sensory inputs to guide ongoing processing. What sort of representation might accomplish this integration? We propose that these representations are *event models*.

## 4 Event models in naturalistic comprehension

Event models are multimodal representations of events that bring together information about people and objects, as well as sequences of actions and their consequences in a spatiotemporal framework (Radvansky & Zacks, 2014). Of particular significance here are the event models that represent events one is currently participating in, sometimes referred to as *working models*, a major function of which is to facilitate prediction‐making to guide perceptions and behaviors^1^ (Eisenberg, Zacks, & Flores, 2018; Zacks, Kurby, Eisenberg, & Haroutunian, 2011). In naturalistic comprehension, the updating of event models occurs at the transition from one event to the next and is associated with widespread shifts in cortical activity patterns and phasic increases in cortical activity. Such neural responses to event boundaries occur in a variety of brain regions—including the PCC—both during movie viewing (Kurby & Zacks, 2018; Zacks et al., 2001; Zacks, Speer, Swallow, & Maley, 2010) and reading or listening to narratives (Speer, Zacks, & Reynolds, 2007; Whitney et al., 2009). In addition, more salient boundaries between events, indexed by greater segmentation agreement across observers, are associated with larger phasic increases in hippocampal activity (Ben‐Yakov & Henson, 2018). The magnitude of these phasic increases reflects successful encoding on a subsequent memory test (Ben‐Yakov & Dudai, 2011) and correlates with activity pattern shifts in the PCC and other posterior DN regions (Baldassano et al., 2017). These results are consistent with the view that PCC and hippocampal interactions serve to integrate recent experiences for episodic encoding (Ranganath & Ritchey, 2012; for a neurocognitive account of hippocampal activity at event‐boundaries, see also Bilkey & Jensen, this volume).

Although the transition between event models involves large cortical responses, particularly in the PCC, that are accompanied by phasic hippocampal activations, initial data investigating the involvement of the midline DN core in the *construction* of events provided mixed support. In a meta‐analysis comparing event model construction in reading situations to a variety of control conditions (Ferstl, Neumann, Bogler, & von Cramon, 2008), activity in the PCC and mPFC was upregulated by event model construction. However, not all findings were consistent with these results. In some studies, PCC activity decreased, rather than increased, in conditions associated with event model construction (e.g, Yarkoni, Speer, & Zacks, 2008; see also Zacks & Ferstl, 2015), and event model construction was also associated with responses in a dorsal mPFC region that is mostly associated with mentalizing and distinct from the midline core mPFC (see Fig. 2; Andrews‐Hanna et al., 2010). These discrepancies challenge a simple account of building event models through increased PCC activity.

Stronger evidence for midline DN involvement in the integration and maintenance of event models comes from studies that scrambled movies or stories at different time scales, from seconds to minutes, and examined in which areas the fMRI response at a given time point was disrupted if what came before was scrambled (Hasson, Yang, Vallines, Heeger, & Rubin, 2008). Using this novel technique, Hasson and colleagues showed in a series of experiments that responses in the midline DN core, and especially the PCC, depended on what had occurred minutes before, whereas responses in primary auditory and visual cortex are essentially unaffected by scrambling the preceding inputs (Hasson, Chen, & Honey, 2015; Hasson et al., 2008; Lerner, Honey, Silbert, & Hasson, 2011). In a follow‐up study, they found that, when listening to intact but not scrambled paragraphs of narratives, the time course of PCC activity in individual subjects tracked the time course of other DN nodes in other subjects—in other words, the time courses were similar across both subjects and regions. Furthermore, the story segments where these correlations were the strongest were also those that were better remembered in a post‐listening memory tests, underscoring the behavioral relevance of this stimulus‐locked functional connectivity of the PCC to event comprehension (Simony et al., 2016). Another study found that cross–subject correlations in DN regions while viewing the beginning of a 10‐minute movie clip were only present when contextual information was provided beforehand; this suggests that the midline DN core is involved in the integration of current sensory inputs with past representations to build event models (Chen et al., 2016). Finally, these researchers also found that the connectivity pattern of the PCC for scrambled movies could reach the level of that of the intact movies when the scrambled segments were presented repeatedly in a fixed order but not when the presentation order was randomly changing with each repetition (Aly, Chen, Turk‐Browne, & Hasson, 2018). These latter findings suggest that, with repeated viewing involving a stable temporal structure, episodic memory can provide context to guide ongoing processing.

**Figure 3:**
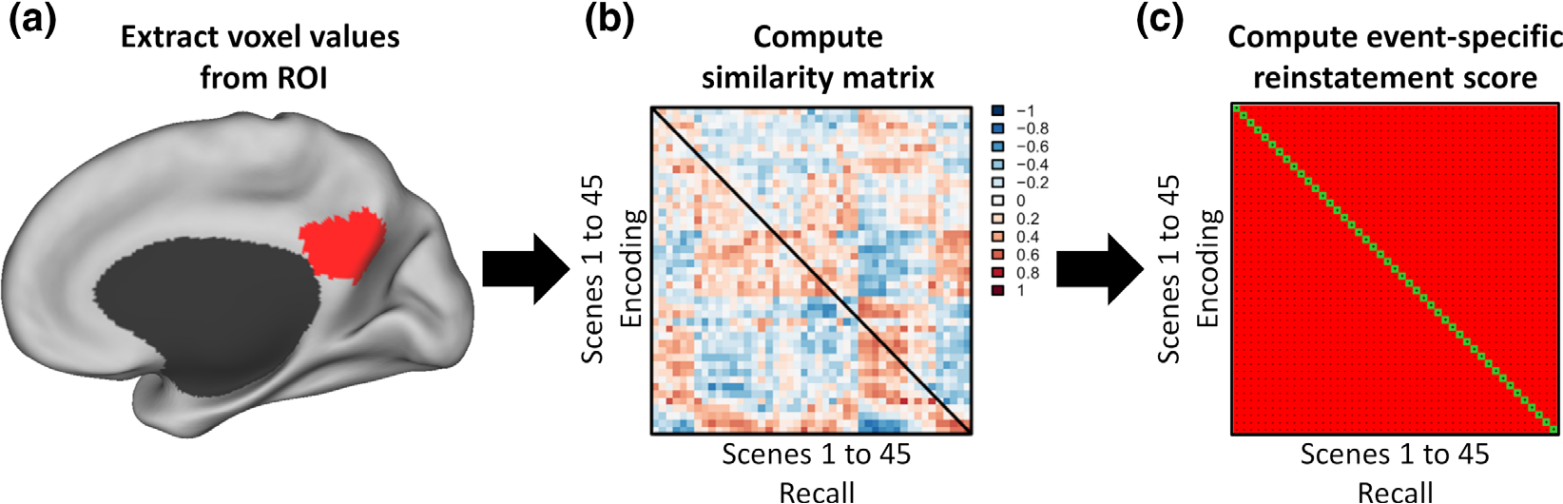
Schematic representation of the analytic procedure involved in using representational similarity analysis to measure memory reinstatement from movies. Following preprocessing, brain activity in regions of interest (ROIs) is averaged per voxel over time, resulting in a single brain image representing the overall brain activity for each individual scene, at both encoding and recall. For instance, illustrated in panel (a) is the PCC ROI, where pattern reinstatement was the highest in Oedekoven et al. (2017). Next, voxel values in the ROI for each scene during encoding are correlated with the corresponding voxel values for all the scenes during recall. The resulting correlation coefficients can then be converted into a normal distribution (with scores ranging from −1 to 1) using Fischer’s *r* to *z* transformation and plotted in a similarity matrix. Panel (b) illustrates a 45 × 45 similarity matrix (based on made‐up data suggestive of a strong memory reinstatement), in which each cell represents a correlation coefficient. Values on the diagonal represent the correlations between brain activity during encoding and recall of the same scene while non‐diagonal values indicate correlations between different scenes. Event‐specific reinstatement scores can then finally be computed, as illustrated in panel (c), by calculating the differences between the diagonal values indicated in green and non‐diagonal values indicated in red. Event‐specific scores that are significantly greater than zero indicate a pattern reinstatement during recall of the brain activity during encoding for the ROI considered. The PCC ROI in panel (a). is displayed on the PALS‐B21 inflated surface map using CARET software (Van Essen, 2005).

In addition to cross–subject correlational studies, recent studies using representational similarity analysis to measure the degree to which brain regions represent event‐specific information have also provided strong evidence for the involvement of the PCC in event models. In two recent studies (Bird, Keidel, Ing, Horner, & Burgess, 2015; Oedekoven, Keidel, Berens, & Bird, 2017), participants were asked to watch and later remember a series of short video clips during fMRI scanning. Event‐specific memory retrieval was operationalized as the degree to which the activity pattern in a brain region during retrieval of an individual clip was more similar to its pattern during viewing this particular clip compared to during viewing all the other clips (see Fig. 3 for a more detailed description of this procedure). Pattern reinstatement was strongest in the PCC. In addition, the degree of reinstatement predicted the amount of detail that was recalled in a subsequent recall test a week later, and also predicted self‐rated memory vividness (for other fMRI studies involving the PCC in event‐specific representations, see Baldassano et al., 2017; Chen et al., 2017; Zadbood, Chen, Leong, Norman, & Hasson, 2017). It is worth noting that stimuli‐matched patterns of brain activity within the PCC and DN in these studies were present even as the activity level in these regions decreased while viewing the videos when compared to rest (Bird et al., 2015). These findings are congruent with functional connectivity studies which show that, even when the PCC is deactivated compared to rest or low demand tasks, it can still show increased functional connectivity with task‐relevant regions (Krieger‐Redwood et al., 2016) and that the strength of this connectivity can predict better task performance (Vatansever, Manktelow, Sahakian, Menon, & Stamatakis, 2018). These findings may help to explain the above‐mentioned inconsistencies in PCC activation level associated with event model construction across reading studies using general linear modeling rather than representational similarity analysis (Zacks & Ferstl, 2015).

Interestingly, these pattern reinstatements effects in the midline DN core seem to be largely insensitive to the sensory modalities involved. For instance, a recent study (Zadbood et al., 2017) investigated activity patterns in the DN and did so not only when participants viewed and then verbally recalled movie scenes (similar to previous studies such as Bird et al., 2015; Chen et al., 2017; Oedekoven et al., 2017), but also when a second group of participants who did not see the movies listened to the spoken recall of the first group. The authors found that the event‐specific patterns in the midline DN core observed in participants who watched the movie scenes were significantly correlated with those of the naïve participants who listened to the descriptions of the scenes. These results suggest that the mental representations of events in the DN midline core do not depend on the sensory modalities in which events are perceived and involve processes that are (at least to some extent) shared across individuals. These results fit well with (a) the above‐mentioned results of increases in PCC activity at event boundaries that are independent of the sensory modality of the considered material (e.g., Speer et al., 2007; Zacks et al., 2001); (b) other fMRI studies showing similar brain responses in the midline DN core when people are being presented with the same narrative across different modalities (e.g., Regev, Honey, Simony, & Hasson, 2013; Tikka, Kauttonen, & Hlushchuk, 2018; Yuan, Major‐Girardin, & Brown, 2018); and (c) behavioral studies showing that the perception of event structure is quite consistent across modalities (e.g., Huff et al., 2018; Magliano, Kopp, McNerney, Radvansky, & Zacks, 2012).

Is the midline DN core the only candidate for representing event models? In a review that largely predated pattern‐based and resting‐state, correlation‐based fMRI, Zacks, Speer, Swallow, Braver, and Reynolds (2007) proposed that the *lateral* PFC was a strong candidate for event model maintenance. Localization to the PFC was supported by data from neurological disease and injury, and early fMRI results suggested that the mPFC might be specialized for event schemas, whereas the lateral PFC might be specialized for event models. In contrast to event models, which represent specific situations strongly anchored in space and time, event schemas are more abstract and semantic representations composed of the stereotyped knowledge that define well‐known situations (Baldassano, Hasson, & Norman, 2018; Zacks et al., 2007). However, the subsequent network‐ and pattern‐based analyses have provided extensive support for the involvement of the midline DN core in event model maintenance, but not the lateral PFC (e.g., Baldassano et al., 2017; Bird et al., 2015; Oedekoven et al., 2017).

In sum, results from studies using naturalistic stimuli show that, rather than specifically subserving interfering self‐generated thoughts, activity in the midline DN core likely reflects the integration of incoming sensory information with prior memory representations to create and maintain coarse event models over relatively extended time scales (e.g., Chen et al., 2016). A major purpose of such event models is to guide the interpretation of novel information. Because they form stable representations of a current event, they also form a basis for subsequent memory. When a specific previously experienced event is retrieved on the basis of external cues (as in the above‐mentioned memory tasks for naturalistic stimuli), a new event model is constructed that tends to be similar to the one that was formed during the encoding experience. These findings fit well with those reviewed in the previous section—primarily that the midline DN core is involved in (re)implementing switching task rules to guide ongoing performance (for a related proposal, see Smith et al., 2018). However, a major difference between comprehending naturalistic events and most laboratory tasks is that naturalistic activities allow one to bring rich prior knowledge to bear, whereas most laboratory tasks are arbitrary by design. Therefore, several repetitions are probably required to build event models from the regularities of laboratory tasks that do not match everyday situations, as shown in Aly et al. (2018) in their study involving repeated presentations of scrambled movies. In the next section, we discuss the processes by which people learn such regularities and relate them to the dominant view, which links the activity of the DN and PCC to self‐generated thoughts and internal mentation.

## 5 Behavioral guidance and self‐generated thoughts

The view that the DN supports combining current information with internally generated representations fits with long‐standing proposals that this network may be specialized for predictive processing. Such claims were initially made to explain the brain’s high energy consumption at rest, suggesting that the brain can be considered as a Bayesian inference engine (e.g., Raichle, 2006; Raichle & Gusnard, 2005; see also Clark, 2013; Hohwy, 2012). A major function of the brain, and particularly the DN, would be to combine external and internal information to update representations of prior knowledge based on stable environmental information; these updated representations would then be used to interpret sensory inputs and guide future behaviors (for a computational account of this proposal using Markov decision processes, see Dohmatob, Dumas, & Bzdok, 2017; see also Pearson et al., 2011). Our proposal is similar to these accounts and suggests that the midline DN is involved in integrating information to guide behavior at the highest level of representation (i.e., event models) over time scales of seconds to minutes, and that such processing operates during both cognitive tasks and during naturalistic perception and action.^2^

Among the theoretical predictive brain accounts, the proposal that the DN supports prediction through the construction of event models is particularly close to the *proactive brain* proposal of Moshe Bar (2007, 2009) and the *situated conceptualization model* of Barsalou (2003, 2009, 2016). Bar’s proposal is mainly based on the findings that the perception of objects that are strongly associated with specific contexts is associated with widespread activity within the midline DN core and parahippocampal cortex compared to objects that are not tied to any particular context (e.g., a toothbrush versus a Rubik's Cube; Bar, 2004). Briefly summarized, Bar proposed that this widespread DN activation corresponds to the activation of contextual information stored in memory that is associated with the perceived object (e.g., for a toothbrush it could be a sink, teeth, toothpaste, etc.). The purpose of this widespread activation would be to form predictions which would then facilitate perception and behavior. Although mostly based on object recognition experiments, Bar suggested that his model also applies to naturalistic situations and proposed that complex inputs can trigger multiple contextual association processes in parallel to generate compound predictions (Bar, 2009; see also Livne & Bar, 2016). Our proposal agrees with this statement, and we suggest that these compound predictions likely correspond to event models that are supported by the PCC and mPFC.

Similar to Bar’s proactive brain proposal, a core aspect of the situated conceptualization model of Barsalou (2003, 2009, 2016) is that concepts (e.g., BICYCLE) do not exist in isolation but are instead typically situated in background settings and are associated with relevant contextual information, resulting in multi‐modal representations of integrated situations. In support of this proposal, a recent neuroimaging study notably showed that the episodic retrieval of previously presented items is associated with the reinstatement in the PCC, precuneus, and angular gyrus of both the activity pattern associated with the item itself and the context in which the item was experienced (Jonker, Dimsdale‐Zucker, Ritchey, Clarke, & Ranganath, 2018). Barsalou proposes that, when a novel situation is encountered in daily life, inferences based on sensory inputs activate the most relevant situated conceptualizations in memory via pattern completion processes (i.e., “What is the perceived situation most like?”). The results are expressed in *simulations* that, at the highest level of representations, are akin to the concept of working event models discussed above. Once situated conceptualizations become active in a simulation, they offer a rich source of predictions about what might occur next. We propose that the DN—and particularly the PCC—might be the key areas where the linking of incoming sensory information with situated conceptualizations take place to form stable event models of the current situation.

The speculative proposal that the midline DN core supports pattern completion inferences based on situated conceptualizations may explain the role of the DN in self‐generated thought. Self‐generated thoughts such as mind‐wandering (Maillet, Seli, & Schacter, 2017), as well as spontaneous memories and future thoughts (Berntsen, 2010, 2018) are often involuntarily triggered by environmental cues and are heavily reliant on the PCC and other DN regions—similar to controlled internal mentation (e.g., Hall et al., 2014). Further, it has been proposed that deliberate self‐generated thoughts might represent an evolutionary adaptation in which the reactivation of past knowledge to construct mental representations of events (past, future, hypothetical, counterfactual, etc.) has become subject to attentional control, rather than being automatically triggered from environmental contingencies (Rasmussen & Bernsten, 2009). We propose here that the deliberate or involuntary entry of self‐generated thoughts into consciousness corresponds to extreme cases of the same integrative processes by which incoming sensory information is associated with memory representations to form event models, the difference being that the activated representations become the main focus of attention rather than supporting attention to the external world. A main purpose of this internal cognition would still be to guide behaviors and allow the making of predictions tough. It has indeed been proposed that a core function of long‐term memory‐based cognition, including mind‐wandering, might be prediction‐making (Bar, Aminoff, Mason, & Fenske, 2007; Schacter et al., 2012; Stawarczyk, Majerus, Maj, Van der Linden, & D’Argembeau, 2011).

More generally, it is possible that activity in the midline DN core fluctuates between different states located on a continuum. At one end of this continuum, we propose that midline DN core activity is the lowest when performing activities where no regularities can be extracted from the environment and where adequate behaviors rely solely upon the processing of current sensory information. This state would, for instance, correspond to the faster RTs with low activity in the midline DN core subsystem during stimulus classification without memory updating (e.g., Smallwood et al., 2013). Although it is likely that the integration of current sensory inputs with memory representations to form event models is still occurring, these event models are not supporting performance to the behavioral task. At an intermediate level, when performing activities that can benefit from predictions based on internal representations of recent events, perception and behavior may rely on midline DN activity to be performed/interpreted accurately. Beneficial aspects of this situation are illustrated by the above examples of midline DN involvement in the comprehension of narrative and movies (e.g., Chen et al., 2016), and by instances of task performance correlating positively with midline DN core activity when responses to external stimuli can be guided by internal representations of task sets and previous trials (e.g., Kucyi, Hove, Esterman, Hutchison, & Valera, 2017; Murphy et al., 2019; Vatansever et al., 2017). Finally, the highest level of PCC activity would occur when attention is redirected to internal representations. This redirection of attentional focus can occur involuntarily, for instance, when memories of past experiences are triggered by perceptual inputs, or through similar evocation of mind‐wandering or future thinking; it can also occur effortfully when individuals deliberately engage in the retrieval and manipulation of past event models to answer environmental demands. When occurring during the performance of tasks that require the constant processing of external stimuli and that do not benefit from memory‐based predictions, such internal mentation would be associated with decreased performance, because their occurrence would likely disrupt attention to the task at hand (Smallwood & Schooler, 2006; Stawarczyk, Majerus, Maquet, et al., 2011; Weissman et al., 2006).

Preliminary support for this proposal comes from a study that examined the neural correlates of ongoing conscious experience during the Sustained Attention to Response Task, a task in which correct responses must constantly be evaluated based on current sensory inputs (Stawarczyk, Majerus, Maquet, et al., 2011). Reporting a complete focus on the ongoing stimuli was associated with the lowest level of DN activity and highest performance. When participants reported that they were thinking about the task (e.g., their performance, its duration, etc.) or when their attention was diverted from the stimuli by task‐unrelated perceptions (e.g., scanner noises, bodily sensations, etc.)—and thus had their attention relying more strongly on the event model of the current situation—DN activity was intermediate. Finally, the highest level of DN activity was found when participants reported experiencing self‐generated thoughts whose content was both decoupled from the task and the current environment.

Further investigation of this proposal could be made by combining representational similarity analysis (see Fig. 3) with an experience sampling procedure during externally directed tasks in which reliance on internal representations can be harmful to performance (e.g., Tusche, Smallwood, Bernhardt, & Singer, 2014). If PCC activity when participants report being fully focused on‐task supports the event model of the currently perceived situation, then activity patterns in this region should be highly similar across the different on‐task reports, both within and across participants, as in all these cases attention is focused on the exact same task. However, when participants are experiencing mind‐wandering, attention becomes focused internally and the event model of the ongoing situation becomes hijacked by idiosyncratic information from long‐term memory. In situations such as these, unless all participants are continuously thinking about the exact same thing whenever they report experiencing mind‐wandering, patterns of activity in the PCC should be dissimilar across mind‐wandering reports, again both between and within participants. Another possibility would be to examine activity pattern in populations with impaired event comprehension (e.g., Alzeihmer disease patients; Bailey, Kurby, Giovannetti, & Zacks, 2013) and to examine whether disrupted neural activity patterns in the midline DN core during tasks involving naturalistic stimuli can be predicted by difficulties in event comprehension.

## 6 A gradient of abstraction within midline structures

As mentioned earlier, the PCC and the mPFC are both core nodes in the DN (Andrews‐Hanna et al., 2010) and are commonly activated in many situations. However, their functions are by no means identical. For example, both the PCC and mPFC are recruited for self‐referential processing, but posterior regions are more active in mental scene construction (Axelrod, Rees, & Bar, 2017). In contrast, the mPFC may process information at a superordinate/schematic level of knowledge structure that is not tied to spatiotemporal constraints (Gilboa & Marlatte, 2017). In support of this proposal, a recent fMRI study (Martial, Stawarczyk, & D’Argembeau, 2018) investigated the neural correlates of self‐representation in a college student population at the general level (i.e., “in general, I am …”; self‐general condition) or a more temporally and spatially constrained level (i.e., “at the University, I am …”; self‐specific condition). They found that both conditions activated the midline DN core compared to a control task. However, the mPFC was more activated in the self‐general condition, whereas the PCC showed greater activity in the self‐specific condition. These results are congruent with meta‐analytic findings showing that the PCC preferentially processes other‐related information and is involved in contextual information integration, whereas the mPFC is more strongly associated with self‐related and motivational processing (Murray, Debbané, Fox, Bzdok, & Eickhoff, 2015; Murray, Schaer, & Debbané, 2012).

Although the PCC and mPFC may support different cognitive processes, functional coupling between these two regions frequently occurs when new information is integrated with schematic knowledge. For example, van Kesteren et al. (2010) reported an increase in PCC‐mPFC coupling when viewing a film’s conclusion, suggesting schematic reinstatement. Further, this coupling predicted later recall performance (see also Bonasia et al., 2018). They proposed that mPFC aids in selectively encoding schema‐consistent elements of experience while suppressing incongruent representations (van Kesteren, Ruiter, Fernandez, & Henson, 2012). Consistent with this hypothesis, other studies have demonstrated that encoding activity in mPFC increases with the degree of schema congruency (e.g., Brod & Shing, 2018; van Kesteren et al., 2013). In addition, fMRI activity patterns in both the PCC and mPFC were sensitive to schematic event structure as participants experienced naturalistic audiovisual sequences that followed familiar scripts (Baldassano et al., 2018).

In sum, whereas the PCC seems more related to the integration of incoming sensory information with memory representations to form event models, the mPFC seems to process information at a superordinate or schematic level that is less reliant upon spatiotemporal context. Functional connectivity studies have shown that these regions interact in situations where incoming information is congruent with these schematic representations, probably reflecting processes by which schemas help structure the formation of event models.

## 7 Conclusion

The midline DN core may be a key resource supporting the establishment and maintenance of working event models, which integrate incoming sensory inputs with past memory representations into coherent representations of “what is happening now.” According to our speculative proposal, a major function of event models is to support predictions about the immediate situation, which facilitates the interpretation of the ongoing activity and guides future behaviors. Importantly, such integration operates both during naturalistic activities and cognitive tasks, which explains why PCC activity is related to better performance in tasks where stimuli processing depends on mental representations of prior trials or known task rules. Self‐generated thoughts may represent extreme instances of this ongoing mechanism, when event model construction is triggered by automatic associative retrieval or by deliberate strategic mechanisms and becomes the center of attentional focus rather than supporting attention to the external world. Within the midline DN subnetwork, the PCC and mPFC are tightly coupled but play slightly differing roles. They coordinate most strongly when higher‐order schematic knowledge—supported primarily by the mPFC—facilitates event model formation supported primarily by the PCC. Finally, the complex integration process to form event models may go on continuously, even in states of functional deactivation compared to rest and low‐demand tasks. Midline DN activity level reflects the extent to which attention relies on these models for content and guidance, which may explain why these regions show the highest basal energy consumption, are major hubs within the DN and, in the case of the PCC, also show dynamic pattern of functional connectivity characterized by transient periods of positive correlations with other cortical brain networks.

## Notes

It should be noted that predictions are probably not the only kind of inferences supported by event models. For instance, recent studies suggest that they also support the formation of backward inferences that help to interpret parts of events that are missed during perception (e.g., Papenmeier, Brockhoff, & Huff, 2019).

It should be noted that the predictions based on events models discussed here are certainly not the only kind of predictions generated by the brain. It is highly probable that other forms of predictions are generated in parallel at faster (and also perhaps slower) timescales, including rapid predictive processing in sensory–motor systems (e.g., Friston, Rosch, Parr, Price, & Bowman, 2017; Kahl & Kopp, 2018).

## References

[tops12450-bib-0001] Aly, M. , Chen, J. , Turk‐Browne, N. B. , & Hasson, U. (2018). Learning naturalistic temporal structure in the posterior medial network. Journal of Cognitive Neuroscience, 30(9), 1345–1365. 10.1162/jocn_a_01308. 30004848 PMC6211568

[tops12450-bib-0002] Andreasen, N. C. , O’Leary, D. S. , Cizadlo, T. , Arndt, S. , Rezai, K. , Watkins, G. L. , Ponto, L. L. B. , & Hichwa, R. D. (1995). Remembering the past: Two facets of episodic memory explored with positron emission tomography. The American Journal of Psychiatry, 152(11), 1576–1585. (7485619). 7485619 10.1176/ajp.152.11.1576

[tops12450-bib-0003] Andrews‐Hanna, J. R. , Reidler, J. S. , Sepulcre, J. , Poulin, R. , & Buckner, R. L. (2010). Functional‐anatomic fractionation of the brain’s default network. Neuron, 65(4), 550–562. (20188659). 20188659 10.1016/j.neuron.2010.02.005 PMC2848443

[tops12450-bib-0004] Andrews‐Hanna, J. R. , Smallwood, J. , & Spreng, R. N. (2014). The default network and self‐generated thought: Component processes, dynamic control, and clinical relevance. Annals of the New York Academy of Sciences, 1316, 29–52. 10.1111/nyas.12360 24502540 PMC4039623

[tops12450-bib-0005] Axelrod, V. , Rees, G. , & Bar, M. (2017). The default network and the combination of cognitive processes that mediate self‐generated thought. Nature Human Behaviour, 1(12), 896–910. 10.1038/s41562-017-0244-9 PMC6054300 30035236

[tops12450-bib-0006] Bailey, H. R. , Kurby, C. A. , Giovannetti, T. , & Zacks, J. M. (2013). Action perception predicts action performance. Neuropsychologia, 51(11), 2294–2304. 10.1016/j.neuropsychologia.2013.06.022 23851113 PMC3936325

[tops12450-bib-0007] Baldassano, C. , Chen, J. , Zadbood, A. , Pillow, J. W. , Hasson, U. , & Norman, K. A. (2017). Discovering event structure in continuous narrative perception and memory. Neuron, 95(3), 709–721.e5. 10.1016/j.neuron.2017.06.041 28772125 PMC5558154

[tops12450-bib-0008] Baldassano, C. , Hasson, U. , & Norman, K. A. (2018). Representation of real‐world event schemas during narrative perception. Journal of Neuroscience, 38(45), 9689–9699. 10.1523/JNEUROSCI.0251-18.2018 30249790 PMC6222059

[tops12450-bib-0009] Bar, M. (2004). Visual objects in context. Nature Reviews Neuroscience, 5(8), 617–629. 10.1038/nrn1476 15263892

[tops12450-bib-0010] Bar, M. (2007). The proactive brain: Using analogies and associations to generate predictions. Trends in Cognitive Sciences, 11(7), 280–289. 17548232 10.1016/j.tics.2007.05.005

[tops12450-bib-0011] Bar, M. (2009). The proactive brain: Memory for predictions. Philosophical Transactions of the Royal Society of London. Series B, Biological Sciences, 364(1521), 1235–1243. 19528004 10.1098/rstb.2008.0310 PMC2666710

[tops12450-bib-0012] Bar, M. , Aminoff, E. , Mason, M. F. , & Fenske, M. (2007). The units of thought. Hippocampus, 17(6), 420–428. 17455334 10.1002/hipo.20287

[tops12450-bib-0013] Barsalou, L. W. (2003). Situated simulation in the human conceptual system. Language and Cognitive Processes, 18(5–6), 513–562. 10.1080/01690960344000026

[tops12450-bib-0014] Barsalou, L. W. (2009). Simulation, situated conceptualization, and prediction. Philosophical Transactions of the Royal Society of London. Series B, Biological Sciences, 364(1521), 1281–1289. 10.1098/rstb.2008.0319 19528009 PMC2666716

[tops12450-bib-0015] Barsalou, L. W. (2016). Situated conceptualization: Theory and applications. In Y. Coello & M. H. Fischer (Eds.), Foundations of embodied cognition: Perceptual and emotional embodiment (pp. 11–37). New York, NY: Routledge/Taylor & Francis Group.

[tops12450-bib-0016] Benoit, R. G. , & Schacter, D. L. (2015). Specifying the core network supporting episodic simulation and episodic memory by activation likelihood estimation. Neuropsychologia, 75, 450–457. 10.1016/j.neuropsychologia.2015.06.034 26142352 PMC4546530

[tops12450-bib-0017] Ben‐Yakov, A. , & Dudai, Y. (2011). Constructing realistic engrams: Poststimulus activity of hippocampus and dorsal striatum predicts subsequent episodic memory. The Journal of Neuroscience, 31(24), 9032–9042. 10.1523/JNEUROSCI.0702-11.2011 21677186 PMC6622928

[tops12450-bib-0018] Ben‐Yakov, A. , & Henson, R. (2018). The hippocampal film‐editor: sensitivity and specificity to event boundaries in continuous experience. Journal of Neuroscience, 38(47), 10057–10068. 10.1523/JNEUROSCI.0524-18.2018 30301758 PMC6246887

[tops12450-bib-0019] Berntsen, D. (2010). The unbidden past: Involuntary autobiographical memories as a basic mode of remembering. Current Directions in Psychological Science, 19(3), 138–142. 10.1177/0963721410370301

[tops12450-bib-0020] Berntsen, D. (2018). Spontaneous future cognitions: An integrative review. Psychological Research Psychologische Forschung, 83(4), 651–665. 10.1007/s00426-018-1127-z 30535833

[tops12450-bib-0021] Bilkey, D. K. , & Jensen, C. (this volume). Neural markers of event boundaries. Topics in Cognitive Science. 10.1111/tops.12470 31621199

[tops12450-bib-0022] Binder, J. R. , Frost, J. A. , Hammeke, T. A. , Bellgowan, P. S. , Rao, S. M. , & Cox, R. W. (1999). Conceptual processing during the conscious resting state. A functional MRI study. Journal of Cognitive Neuroscience, 11(1), 80–93. (9950716). 9950716 10.1162/089892999563265

[tops12450-bib-0023] Bird, C. M. , Keidel, J. L. , Ing, L. P. , Horner, A. J. , & Burgess, N. (2015). Consolidation of complex events via reinstatement in posterior cingulate cortex. Journal of Neuroscience, 35(43), 14426–14434. 10.1523/JNEUROSCI.1774-15.2015 26511235 PMC4623223

[tops12450-bib-0024] Bonasia, K. , Sekeres, M. J. , Gilboa, A. , Grady, C. L. , Winocur, G. , & Moscovitch, M. (2018). Prior knowledge modulates the neural substrates of encoding and retrieving naturalistic events at short and long delays. Neurobiology of Learning and Memory, 153, 26–39. 10.1016/j.nlm.2018.02.017 29474955

[tops12450-bib-0025] Brod, G. , & Shing, Y. L. (2018). Specifying the role of the ventromedial prefrontal cortex in memory formation. Neuropsychologia, 111, 8–15. 10.1016/j.neuropsychologia.2018.01.005 29317324

[tops12450-bib-0026] Brodmann, K. (1909). Vergleichende Lokalisationslehre der Großhirnrinde: in ihren Prinzipien dargestellt auf Grund des Zellenbaues. Leipzig: Johann Ambrosius Barth.

[tops12450-bib-0027] Buckner, R. L. , & Carroll, D. C. (2007). Self‐projection and the brain. Trends in Cognitive Sciences, 11(2), 49–57. (17188554). 17188554 10.1016/j.tics.2006.11.004

[tops12450-bib-0028] Buckner, R. L. , Sepulcre, J. , Talukdar, T. , Krienen, F. M. , Liu, H. , Hedden, T. , & Johnson, K. A. (2009). Cortical hubs revealed by intrinsic functional connectivity: Mapping, assessment of stability, and relation to Alzheimer’s disease. The Journal of Neuroscience, 29(6), 1860–1873. (Peer Reviewed Journal: 2009–02211‐035). 19211893 10.1523/JNEUROSCI.5062-08.2009 PMC2750039

[tops12450-bib-0029] Bzdok, D. , Heeger, A. , Langner, R. , Laird, A. R. , Fox, P. T. , Palomero‐Gallagher, N. , Vogt, B. A. , Zilles, K. , & Eickhoff, S. B. (2015). Subspecialization in the human posterior medial cortex. NeuroImage, 106, 55–71. 10.1016/j.neuroimage.2014.11.009 25462801 PMC4780672

[tops12450-bib-0030] Chen, J. , Honey, C. J. , Simony, E. , Arcaro, M. J. , Norman, K. A. , & Hasson, U. (2016). Accessing real‐life episodic information from minutes versus hours earlier modulates hippocampal and high‐order cortical dynamics. Cerebral Cortex, 26(8), 3428–3441. 10.1093/cercor/bhv155 26240179 PMC4961013

[tops12450-bib-0031] Chen, J. , Leong, Y. C. , Honey, C. J. , Yong, C. H. , Norman, K. A. , & Hasson, U. (2017). Shared memories reveal shared structure in neural activity across individuals. Nature Neuroscience, 20(1), 115–125. 10.1038/nn.4450 27918531 PMC5191958

[tops12450-bib-0032] Christoff, K. , Gordon, A. M. , Smallwood, J. , Schooler, J. W. , & Smith, R. (2009). Experience sampling during fMRI reveals default network and executive system contributions to mind wandering. Proceedings of the National Academy of Sciences of the United States of America, 106(21), 8719–8724. 10.1073/pnas.0900234106 19433790 PMC2689035

[tops12450-bib-0033] Christoff, K. , Irving, Z. C. , Fox, K. C. , Spreng, R. N. , & Andrews‐Hanna, J. R. (2016). Mind‐wandering as spontaneous thought: A dynamic framework. Nature Reviews Neuroscience, 17(11), 718–731. 10.1038/nrn.2016.113 27654862

[tops12450-bib-0034] Chun, M. M. , Golomb, J. D. , & Turk‐Browne, N. B. (2011). A taxonomy of external and internal attention. Annual Review of Psychology, 62(1), 73–101. 10.1146/annurev.psych.093008.100427 19575619

[tops12450-bib-0035] Clark, A. (2013). Whatever next? Predictive brains, situated agents, and the future of cognitive science. Behavioral and Brain Sciences, 36(3), 181–204. 10.1017/S0140525X12000477 23663408

[tops12450-bib-0036] Córcoles‐Parada, M. , Müller, N. C. J. , Ubero, M. , Serrano‐Del‐Pueblo, V. M. , Mansilla, F. , Marcos‐Rabal, P. , Artacho‐Pérula, E. , Dresler, M. , Insausti, R. , Fernández, G. , & Muñoz‐López, M. (2017). Anatomical segmentation of the human medial prefrontal cortex. The Journal of Comparative Neurology, 525(10), 2376–2393. 10.1002/cne.24212 28317116

[tops12450-bib-0037] Crittenden, B. M. , Mitchell, D. J. , & Duncan, J. (2015). Recruitment of the default mode network during a demanding act of executive control. eLife, 4, e06481. 10.7554/eLife.06481 25866927 PMC4427863

[tops12450-bib-0038] D’Argembeau, A. (2013). On the role of the ventromedial prefrontal cortex in self‐processing: The valuation hypothesis. Frontiers in Human Neuroscience, 7, 372. 10.3389/fnhum.2013.00372 23847521 PMC3707083

[tops12450-bib-0039] de Pasquale, F. , Corbetta, M. , Betti, V. , & Della Penna, S. (2018). Cortical cores in network dynamics. NeuroImage, 180, 370–382. 10.1016/j.neuroimage.2017.09.063 28974453

[tops12450-bib-0040] de Pasquale, F. , Della Penna, S. , Sporns, O. , Romani, G. L. , & Corbetta, M. (2016). A dynamic core network and global efficiency in the resting human brain. Cerebral Cortex, 26(10), 4015–4033. 10.1093/cercor/bhv185 26347485 PMC5027996

[tops12450-bib-0041] Dixon, M. L. , Andrews‐Hanna, J. R. , Spreng, R. N. , Irving, Z. C. , Mills, C. , Girn, M. , & Christoff, K. (2017). Interactions between the default network and dorsal attention network vary across default subsystems, time, and cognitive states. NeuroImage, 147, 632–649. 10.1016/j.neuroimage.2016.12.073 28040543

[tops12450-bib-0042] Dixon, M. L. , Fox, K. C. , & Christoff, K. (2014). A framework for understanding the relationship between externally and internally directed cognition. Neuropsychologia, 62, 321–330. 10.1016/j.neuropsychologia.2014.05.024 24912071

[tops12450-bib-0043] Dohmatob, E. , Dumas, G. , & Bzdok, D. (2017). Dark control: A unified account of default mode function by control theory and reinforcement learning. BioRxiv, 148890. 10.1101/148890

[tops12450-bib-0044] Eisenberg, M. L. , Zacks, J. M. , & Flores, S. (2018). Dynamic prediction during perception of everyday events. Cognitive Research: Principles and Implications, 3(1), 53. 10.1186/s41235-018-0146-z 30594977 PMC6311167

[tops12450-bib-0045] Ferstl, E. C. , Neumann, J. , Bogler, C. , & von Cramon, D. Y. (2008). The extended language network: A meta‐analysis of neuroimaging studies on text comprehension. Human Brain Mapping, 29(5), 581–593. 17557297 10.1002/hbm.20422 PMC2878642

[tops12450-bib-0046] Fox, M. D. , & Raichle, M. E. (2007). Spontaneous fluctuations in brain activity observed with functional magnetic resonance imaging. Nature Reviews Neuroscience, 8(9), 700–711. 10.1038/nrn2201 17704812

[tops12450-bib-0047] Fox, M. D. , Snyder, A. Z. , Vincent, J. L. , Corbetta, M. , Van Essen, D. C. , & Raichle, M. E. (2005). The human brain is intrinsically organized into dynamic, anticorrelated functional networks. PNAS: Proceedings of the National Academy of Sciences of the United States of America, 102(27), 9673–9678. (Peer Reviewed Journal: 2007–01782‐001). 10.1073/pnas.0504136102 PMC1157105 15976020

[tops12450-bib-0048] Friston, K. J. , Rosch, R. , Parr, T. , Price, C. , & Bowman, H. (2017). Deep temporal models and active inference. Neuroscience & Biobehavioral Reviews, 77, 388–402. 10.1016/j.neubiorev.2017.04.009 28416414 PMC5461873

[tops12450-bib-0049] Gilboa, A. , & Marlatte, H. (2017). Neurobiology of schemas and schema‐mediated memory. Trends in Cognitive Sciences, 21(8), 618–631. 10.1016/j.tics.2017.04.013 28551107

[tops12450-bib-0050] González‐García, C. , Flounders, M. W. , Chang, R. , Baria, A. T. , & He, B. J. (2018). Content‐specific activity in frontoparietal and default‐mode networks during prior‐guided visual perception. eLife, 7, e36068. 10.7554/eLife.36068 30063006 PMC6067880

[tops12450-bib-0051] Greicius, M. D. , Krasnow, B. , Reiss, A. L. , & Menon, V. (2003). Functional connectivity in the resting brain: A network analysis of the default mode hypothesis. Proceedings of the National Academy of Sciences of the United States of America, 100(1), 253–258. (12506194). 12506194 10.1073/pnas.0135058100 PMC140943

[tops12450-bib-0052] Gusnard, D. A. , Akbudak, E. , Shulman, G. L. , & Raichle, M. E. (2001). Medial prefrontal cortex and self‐referential mental activity: Relation to a default mode of brain function. Proceedings of the National Academy of Sciences, 98(7), 4259–4264. (11259662). 10.1073/pnas.071043098 PMC31213 11259662

[tops12450-bib-0053] Gusnard, D. A. , & Raichle, M. E. (2001). Searching for a baseline: functional imaging and the resting human brain. Nature Reviews Neuroscience, 2(10), 685–694. (11584306). 11584306 10.1038/35094500

[tops12450-bib-0054] Hall, S. A. , Rubin, D. C. , Miles, A. , Davis, S. W. , Wing, E. A. , Cabeza, R. , & Berntsen, D. (2014). The neural basis of involuntary episodic memories. Journal of Cognitive Neuroscience, 26(10), 2385–2399. 10.1162/jocn_a_00633 24702453 PMC4149828

[tops12450-bib-0055] Hasson, U. , Chen, J. , & Honey, C. J. (2015). Hierarchical process memory: memory as an integral component of information processing. Trends in Cognitive Sciences, 19(6), 304–313. 10.1016/j.tics.2015.04.006 25980649 PMC4457571

[tops12450-bib-0056] Hasson, U. , Nir, Y. , Levy, I. , Fuhrmann, G. , & Malach, R. (2004). Intersubject synchronization of cortical activity during natural vision. Science, 303(5664), 1634–1640. 10.1126/science.1089506 15016991

[tops12450-bib-0057] Hasson, U. , Yang, E. , Vallines, I. , Heeger, D. J. , & Rubin, N. (2008). A hierarchy of temporal receptive windows in human cortex. Journal of Neuroscience, 28(10), 2539–2550. 10.1523/JNEUROSCI.5487-07.2008 18322098 PMC2556707

[tops12450-bib-0058] Hohwy, J. (2012). Attention and conscious perception in the hypothesis testing brain. Frontiers in Psychology, 3, 96. 10.3389/fpsyg.2012.00096 22485102 PMC3317264

[tops12450-bib-0059] Huff, M. , Maurer, A. E. , Brich, I. , Pagenkopf, A. , Wickelmaier, F. , & Papenmeier, F. (2018). Construction and updating of event models in auditory event processing. Journal of Experimental Psychology: Learning, Memory, and Cognition, 44(2), 307–320. 10.1037/xlm0000482 28933900

[tops12450-bib-0060] Jonker, T. R. , Dimsdale‐Zucker, H. , Ritchey, M. , Clarke, A. , & Ranganath, C. (2018). Neural reactivation in parietal cortex enhances memory for episodically linked information. Proceedings of the National Academy of Sciences, 115(43), 11084–11089. 10.1073/pnas.1800006115 PMC6205442 30297400

[tops12450-bib-0061] Kabbara, A. , Falou, W. E. , Khalil, M. , Wendling, F. , & Hassan, M. (2017). The dynamic functional core network of the human brain at rest. Scientific Reports, 7(1), 2936. 10.1038/s41598-017-03420-6 28592794 PMC5462789

[tops12450-bib-0062] Kahl, S. , & Kopp, S. (2018). A predictive processing model of perception and action for self‐other distinction. Frontiers in Psychology, 9, 2421. 10.3389/fpsyg.2018.02421 30559703 PMC6287016

[tops12450-bib-0063] Kelly, A. M. , Uddin, L. Q. , Biswal, B. B. , Castellanos, F. X. , & Milham, M. P. (2008). Competition between functional brain networks mediates behavioral variability. NeuroImage, 39(1), 527–537. 10.1016/j.neuroimage.2007.08.008 17919929

[tops12450-bib-0064] Kim, H. (2012). A dual‐subsystem model of the brain’s default network: Self‐referential processing, memory retrieval processes, and autobiographical memory retrieval. NeuroImage, 61(4), 966–977. 10.1016/j.neuroimage.2012.03.025 22446489

[tops12450-bib-0065] Kim, H. (2016). Default network activation during episodic and semantic memory retrieval: A selective meta‐analytic comparison. Neuropsychologia, 80, 35–46. 10.1016/j.neuropsychologia.2015.11.006 26562053

[tops12450-bib-0066] Konishi, M. , McLaren, D. G. , Engen, H. , & Smallwood, J. (2015). Shaped by the past: The default mode network supports cognition that is independent of immediate perceptual input. PLoS ONE, 10(6), e0132209. 10.1371/journal.pone.0132209 26125559 PMC4488375

[tops12450-bib-0067] Koshino, H. , Minamoto, T. , Yaoi, K. , Osaka, M. , & Osaka, N. (2014). Coactivation of the default mode network regions and working memory network regions during task preparation. Scientific Reports, 4, 5954. 10.1038/srep05954 25092432 PMC4121601

[tops12450-bib-0068] Krieger‐Redwood, K. , Jefferies, E. , Karapanagiotidis, T. , Seymour, R. , Nunes, A. , Ang, J. W. A. , Majernikova, V. , Mollo, G. , & Smallwood, J. (2016). Down but not out in posterior cingulate cortex: Deactivation yet functional coupling with prefrontal cortex during demanding semantic cognition. NeuroImage, 141, 366–377. 10.1016/j.neuroimage.2016.07.060 27485753 PMC5035136

[tops12450-bib-0069] Kucyi, A. , Hove, M. J. , Esterman, M. , Hutchison, R. M. , & Valera, E. M. (2017). Dynamic brain network correlates of spontaneous fluctuations in attention. Cerebral Cortex, 27(3), 1831–1840. 10.1093/cercor/bhw029 26874182 PMC6317462

[tops12450-bib-0070] Kurby, C. A. , & Zacks, J. M. (2018). Preserved neural event segmentation in healthy older adults. Psychology and Aging, 33(2), 232–245. 10.1037/pag0000226 29446971 PMC8577268

[tops12450-bib-0071] Leech, R. , Braga, R. , & Sharp, D. J. (2012). Echoes of the brain within the posterior cingulate cortex. Journal of Neuroscience, 32(1), 215–222. 10.1523/JNEUROSCI.3689-11.2012 22219283 PMC6621313

[tops12450-bib-0072] Leech, R. , & Sharp, D. J. (2014). The role of the posterior cingulate cortex in cognition and disease. Brain, 137(Pt 1), 12–32. 10.1093/brain/awt162 23869106 PMC3891440

[tops12450-bib-0073] Lerner, Y. , Honey, C. J. , Silbert, L. J. , & Hasson, U. (2011). Topographic mapping of a hierarchy of temporal receptive windows using a narrated story. Journal of Neuroscience, 31(8), 2906–2915. 10.1523/JNEUROSCI.3684-10.2011 21414912 PMC3089381

[tops12450-bib-0074] Livne, T. , & Bar, M. (2016). Cortical integration of contextual information across objects. Journal of Cognitive Neuroscience, 28(7), 948–958. 10.1162/jocn_a_00944 26942322

[tops12450-bib-0075] Magliano, J. , Kopp, K. , McNerney, M. W. , Radvansky, G. A. , & Zacks, J. M. (2012). Aging and perceived event structure as a function of modality. Aging, Neuropsychology, and Cognition, 19(1–2), 264–282. 10.1080/13825585.2011.633159 PMC3347870 22182344

[tops12450-bib-0076] Maillet, D. , Seli, P. , & Schacter, D. L. (2017). Mind‐wandering and task stimuli: Stimulus‐dependent thoughts influence performance on memory tasks and are more often past‐ versus future‐oriented. Consciousness and Cognition, 52, 55–67. 10.1016/j.concog.2017.04.014 28460272 PMC5494999

[tops12450-bib-0077] Margulies, D. S. , Ghosh, S. S. , Goulas, A. , Falkiewicz, M. , Huntenburg, J. M. , Langs, G. , Bezgin, G. , Eickhoff, S. B. , Castellanos, F. X. , Petrides, M. , Jefferies, E. , & Smallwood, J. (2016). Situating the default‐mode network along a principal gradient of macroscale cortical organization. Proceedings of the National Academy of Sciences, 113(44), 12574–12579. 10.1073/pnas.1608282113 PMC5098630 27791099

[tops12450-bib-0078] Margulies, D. S. , & Smallwood, J. (2017). Converging evidence for the role of transmodal cortex in cognition. Proceedings of the National Academy of Sciences, 114(48), 12641–12643. 10.1073/pnas.1717374114 PMC5715795 29142008

[tops12450-bib-0079] Martial, C. , Stawarczyk, D. , & D’Argembeau, A. (2018). Neural correlates of context‐independent and context‐dependent self‐knowledge. Brain and Cognition, 125, 23–31. 10.1016/j.bandc.2018.05.004 29807267

[tops12450-bib-0080] Murphy, C. , Wang, H.‐T. , Konu, D. , Lowndes, R. , Margulies, D. S. , Jefferies, E. , & Smallwood, J. (2019). Modes of operation: A topographic neural gradient supporting stimulus dependent and independent cognition. NeuroImage, 186, 487–496. 10.1016/j.neuroimage.2018.11.009 30447291

[tops12450-bib-0081] Murray, R. J. , Debbané, M. , Fox, P. T. , Bzdok, D. , & Eickhoff, S. B. (2015). Functional connectivity mapping of regions associated with self‐ and other‐processing. Human Brain Mapping, 36(4), 1304–1324. 10.1002/hbm.22703 25482016 PMC4791034

[tops12450-bib-0082] Murray, R. J. , Schaer, M. , & Debbané, M. (2012). Degrees of separation: A quantitative neuroimaging meta‐analysis investigating self‐specificity and shared neural activation between self‐ and other‐reflection. Neuroscience & Biobehavioral Reviews, 36(3), 1043–1059. 10.1016/j.neubiorev.2011.12.013 22230705

[tops12450-bib-0083] Norman, K. , Polyn, S. , Detre, G. , & Haxby, J. (2006). Beyond mind‐reading: Multi‐voxel pattern analysis of fMRI data. Trends in Cognitive Sciences, 10(9), 424–430. 16899397 10.1016/j.tics.2006.07.005

[tops12450-bib-0084] Oedekoven, C. S. H. , Keidel, J. L. , Berens, S. C. , & Bird, C. M. (2017). Reinstatement of memory representations for lifelike events over the course of a week. Scientific Reports, 7(1), 14305. 10.1038/s41598-017-13938-4 29084981 PMC5662713

[tops12450-bib-0085] Papenmeier, F. , Brockhoff, A. , & Huff, M. (2019). Filling the gap despite full attention: The role of fast backward inferences for event completion. Cognitive Research: Principles and Implications, 4(1), 3. 10.1186/s41235-018-0151-2 30693396 PMC6352563

[tops12450-bib-0086] Pearson, J. M. , Heilbronner, S. R. , Barack, D. L. , Hayden, B. Y. , & Platt, M. L. (2011). Posterior cingulate cortex: Adapting behavior to a changing world. Trends in Cognitive Sciences, 15(4), 143–151. (21420893) 21420893 10.1016/j.tics.2011.02.002 PMC3070780

[tops12450-bib-0087] Radvansky, G. A. , & Zacks, J. M. (2014). Event cognition. Oxford, UK: Oxford University Press.

[tops12450-bib-0088] Raichle, M. E. (2006). Neuroscience. The brain’s dark energy. [Erratum appears in Science. 2007 Jan 12;315(5809):187]. Science, 314(5803), 1249–1250. (17124311). 17124311

[tops12450-bib-0089] Raichle, M. E. , & Gusnard, D. A. (2005). Intrinsic brain activity sets the stage for expression of motivated behavior. Journal of Comparative Neurology, 493(1), 167–176. (16254998) 10.1002/cne.20752 16254998

[tops12450-bib-0090] Ranganath, C. , & Ritchey, M. (2012). Two cortical systems for memory‐guided behaviour. Nature Reviews Neuroscience, 13(10), 713–726. 10.1038/nrn3338 22992647

[tops12450-bib-0091] Rasmussen, A. S. , & Bernsten, D. (2009). The possible functions of involuntary autobiographical memories. Applied Cognitive Psychology, 23(8), 1137–1152. 10.1002/acp.1615

[tops12450-bib-0092] Regev, M. , Honey, C. J. , Simony, E. , & Hasson, U. (2013). Selective and invariant neural responses to spoken and written narratives. Journal of Neuroscience, 33(40), 15978–15988. 10.1523/JNEUROSCI.1580-13.2013 24089502 PMC3787506

[tops12450-bib-0093] Richmond, L. L. , & Zacks, J. M. (2017). Constructing experience: Event models from perception to action. Trends in Cognitive Sciences, 21(12), 962–980. 10.1016/j.tics.2017.08.005 28899609 PMC5694361

[tops12450-bib-0094] Schacter, D. L. , Addis, D. R. , Hassabis, D. , Martin, V. C. , Spreng, R. N. , & Szpunar, K. K. (2012). The future of memory: Remembering, imagining, and the brain. Neuron, 76(4), 677–694. 10.1016/j.neuron.2012.11.001 23177955 PMC3815616

[tops12450-bib-0095] Sepulcre, J. , Sabuncu, M. R. , Yeo, T. B. , Liu, H. , & Johnson, K. A. (2012). Stepwise connectivity of the modal cortex reveals the multimodal organization of the human brain. Journal of Neuroscience, 32(31), 10649–10661. 10.1523/JNEUROSCI.0759-12.2012 22855814 PMC3483645

[tops12450-bib-0096] Sestieri, C. , Shulman, G. L. , & Corbetta, M. (2010). Attention to memory and the environment: functional specialization and dynamic competition in human posterior parietal cortex. Journal of Neuroscience, 30(25), 8445–8456. 10.1523/JNEUROSCI.4719-09.2010 20573892 PMC2906749

[tops12450-bib-0097] Sestieri, C. , Shulman, G. L. , & Corbetta, M. (2017). The contribution of the human posterior parietal cortex to episodic memory. Nature Reviews Neuroscience, 18(3), 183–192. 10.1038/nrn.2017.6 28209980 PMC5682023

[tops12450-bib-0098] Shulman, G. L. , Fiez, J. A. , Corbetta, M. , Buckner, R. L. , Miezin, F. M. , Raichle, M. E. , & Petersen, S. E. (1997). Common blood flow changes across visual tasks: II.: Decreases in cerebral cortex. Journal of Cognitive Neuroscience, 9(5), 648–663. 10.1162/jocn.1997.9.5.648 23965122

[tops12450-bib-0099] Simony, E. , Honey, C. J. , Chen, J. , Lositsky, O. , Yeshurun, Y. , Wiesel, A. , & Hasson, U. (2016). Dynamic reconfiguration of the default mode network during narrative comprehension. Nature Communications, 7, 12141. 10.1038/ncomms12141 PMC4960303 27424918

[tops12450-bib-0100] Smallwood, J. , Brown, K. , Baird, B. , & Schooler, J. W. (2012). Cooperation between the default mode network and the frontal‐parietal network in the production of an internal train of thought. Brain Research, 1428, 60–70. (21466793). 21466793 10.1016/j.brainres.2011.03.072

[tops12450-bib-0101] Smallwood, J. , Karapanagiotidis, T. , Ruby, F. , Medea, B. , de Caso, I. , Konishi, M. , Wang, H.‐T. , Hallam, G. , Margulies, D. S. , & Jefferies, E. (2016). Representing representation: Integration between the temporal lobe and the posterior cingulate influences the content and form of spontaneous thought. PLoS ONE, 11(4), e0152272. 10.1371/journal.pone.0152272 27045292 PMC4821638

[tops12450-bib-0102] Smallwood, J. , & Schooler, J. W. (2006). The restless mind. Psychological Bulletin, 132(6), 946–958. 10.1037/0033-2909.132.6.946 17073528

[tops12450-bib-0103] Smallwood, J. , Tipper, C. , Brown, K. , Baird, B. , Engen, H. , Michaels, J. R. , Grafton, S. , & Schooler, J. W. (2013). Escaping the here and now: Evidence for a role of the default mode network in perceptually decoupled thought. NeuroImage, 69, 120–125. 10.1016/j.neuroimage.2012.12.012 23261640

[tops12450-bib-0104] Smith, V. , Mitchell, D. J. , & Duncan, J. (2018). Role of the default mode network in cognitive transitions. Cerebral Cortex, 28(10), 3685–3696. 10.1093/cercor/bhy167 30060098 PMC6132281

[tops12450-bib-0105] Sonuga‐Barke, E. J. , & Castellanos, F. X. (2007). Spontaneous attentional fluctuations in impaired states and pathological conditions: A neurobiological hypothesis. Neuroscience and Biobehavioral Reviews, 31(7), 977–986. 10.1016/j.neubiorev.2007.02.005 17445893

[tops12450-bib-0106] Speer, N. K. , Zacks, J. M. , & Reynolds, J. R. (2007). Human brain activity time‐locked to narrative event boundaries. Psychological Science, 18(5), 449–455. 10.1111/j.1467-9280.2007.01920.x 17576286

[tops12450-bib-0107] Spreng, R. N. (2012). The fallacy of a “task‐negative” network. Frontiers in Psychology, 3, 145. 10.3389/fpsyg.2012.00145 22593750 PMC3349953

[tops12450-bib-0108] Spreng, R. N. , DuPre, E. , Selarka, D. , Garcia, J. , Gojkovic, S. , Mildner, J. , Luh, W.‐M. , & Turner, G. R. (2014). Goal‐congruent default network activity facilitates cognitive control. Journal of Neuroscience, 34(42), 14108–14114. 10.1523/JNEUROSCI.2815-14.2014 25319706 PMC4198547

[tops12450-bib-0109] Spreng, R. N. , Mar, R. A. , & Kim, A. S. (2009). The common neural basis of autobiographical memory, prospection, navigation, theory of mind, and the default mode: A quantitative meta‐analysis. Journal of Cognitive Neuroscience, 21(3), 489–510. 10.1162/jocn.2008.21029 18510452

[tops12450-bib-0110] Stawarczyk, D. , & D’Argembeau, A. (2015). Neural correlates of personal goal processing during episodic future thinking and mind‐wandering: An ALE meta‐analysis. Human Brain Mapping, 36(8), 2928–2947. 10.1002/hbm.22818 25931002 PMC6869624

[tops12450-bib-0111] Stawarczyk, D. , Majerus, S. , Maj, M. , Van der Linden, M. , & D’Argembeau, A. (2011). Mind‐wandering: phenomenology and function as assessed with a novel experience sampling method. Acta Psychologica, 136(3), 370–381. 10.1016/j.actpsy.2011.01.002 21349473

[tops12450-bib-0112] Stawarczyk, D. , Majerus, S. , Maquet, P. , & D’Argembeau, A. (2011). Neural correlates of ongoing conscious experience: Both task‐unrelatedness and stimulus‐independence are related to default network activity. PLoS ONE, 6(2), e16997. 10.1371/journal.pone.0016997 21347270 PMC3038939

[tops12450-bib-0113] Tikka, P. , Kauttonen, J. , & Hlushchuk, Y. (2018). Narrative comprehension beyond language: Common brain networks activated by a movie and its script. PLoS ONE, 13(7), e0200134. 10.1371/journal.pone.0200134 29969491 PMC6029793

[tops12450-bib-0114] Tusche, A. , Smallwood, J. , Bernhardt, B. C. , & Singer, T. (2014). Classifying the wandering mind: Revealing the affective content of thoughts during task‐free rest periods. NeuroImage, 97, 107–116. 10.1016/j.neuroimage.2014.03.076 24705200

[tops12450-bib-0115] van den Heuvel, M. P. , & Sporns, O. (2013). Network hubs in the human brain. Trends in Cognitive Sciences, 17(12), 683–696. 10.1016/j.tics.2013.09.012 24231140

[tops12450-bib-0116] Van Essen, D. C. (2005). A Population‐Average, Landmark‐ and Surface‐based (PALS) atlas of human cerebral cortex. NeuroImage, 28(3), 635–662. 10.1016/j.neuroimage.2005.06.058 16172003

[tops12450-bib-0117] van Kesteren, M. T. R. , Beul, S. F. , Takashima, A. , Henson, R. N. , Ruiter, D. J. , & Fernández, G. (2013). Differential roles for medial prefrontal and medial temporal cortices in schema‐dependent encoding: From congruent to incongruent. Neuropsychologia, 51(12), 2352–2359. 10.1016/j.neuropsychologia.2013.05.027 23770537

[tops12450-bib-0118] van Kesteren, M. T. R. , Fernandez, G. , Norris, D. G. , & Hermans, E. J. (2010). Persistent schema‐dependent hippocampal‐neocortical connectivity during memory encoding and postencoding rest in humans. Proceedings of the National Academy of Sciences, 107(16), 7550–7555. 10.1073/pnas.0914892107 PMC2867741 20363957

[tops12450-bib-0119] van Kesteren, M. T. R. , Ruiter, D. J. , Fernandez, G. , & Henson, R. N. (2012). How schema and novelty augment memory formation. Trends in Neurosciences, 35(4), 211–219. 10.1016/j.tins.2012.02.001 22398180

[tops12450-bib-0120] Vatansever, D. , Manktelow, A. , Sahakian, B. J. , Menon, D. K. , & Stamatakis, E. A. (2018). Default mode network engagement beyond self‐referential internal mentation. Brain Connectivity, 8(4), 245–253. 10.1089/brain.2017.0489 29366339

[tops12450-bib-0121] Vatansever, D. , Menon, D. K. , & Stamatakis, E. A. (2017). Default mode contributions to automated information processing. Proceedings of the National Academy of Sciences, 114(48), 12821–12826. 10.1073/pnas.1710521114 PMC5715758 29078345

[tops12450-bib-0122] Vogt, B. A. (2009). Cingulate neurobiology and disease. Oxford, UK: Oxford University Press.

[tops12450-bib-0123] Weissman, D. H. , Roberts, K. C. , Visscher, K. M. , & Woldorff, M. G. (2006). The neural bases of momentary lapses in attention. Nature Neuroscience, 9(7), 971–978. (16767087) 16767087 10.1038/nn1727

[tops12450-bib-0124] Whitney, C. , Huber, W. , Klann, J. , Weis, S. , Krach, S. , & Kircher, T. (2009). Neural correlates of narrative shifts during auditory story comprehension. NeuroImage, 47(1), 360–366. 10.1016/j.neuroimage.2009.04.037 19376237

[tops12450-bib-0125] Yarkoni, T. , Speer, N. K. , & Zacks, J. M. (2008). Neural substrates of narrative comprehension and memory. NeuroImage, 41(4), 1408–1425. 10.1016/j.neuroimage.2008.03.062 18499478 PMC2580728

[tops12450-bib-0126] Yeo, B. T. , Krienen, F. M. , Sepulcre, J. , Sabuncu, M. R. , Lashkari, D. , Hollinshead, M. , Roffman, J. L. , Smoller, J. W. , Zöllei, L. , Polimeni, J. R. , Fischl, B. , Liu, H. , & Buckner, R. L. (2011). The organization of the human cerebral cortex estimated by intrinsic functional connectivity. Journal of Neurophysiology, 106(3), 1125–1165. 10.1152/jn.00338.2011 21653723 PMC3174820

[tops12450-bib-0127] Yuan, Y. , Major‐Girardin, J. , & Brown, S. (2018). Storytelling is intrinsically mentalistic: A functional magnetic resonance imaging study of narrative production across modalities. Journal of Cognitive Neuroscience, 30(9), 1298–1314. 10.1162/jocn_a_01294 29916789

[tops12450-bib-0128] Zacks, J. M. , Braver, T. S. , Sheridan, M. A. , Donaldson, D. I. , Snyder, A. Z. , Ollinger, J. M. , Buckner, R. L. , & Raichle, M. E. (2001). Human brain activity time‐locked to perceptual event boundaries. Nature Neuroscience, 4(6), 651–655. 11369948 10.1038/88486

[tops12450-bib-0129] Zacks, J. M. , & Ferstl, E. C. (2015). Discourse comprehension. In G. Hickok & S. L. Small (Eds.), Neurobiology of language (pp. 662–674). Amsterdam: Elsevier Science Publishers.

[tops12450-bib-0130] Zacks, J. M. , Kurby, C. A. , Eisenberg, M. L. , & Haroutunian, N. (2011). Prediction error associated with the perceptual segmentation of naturalistic events. Journal of Cognitive Neuroscience, 23(12), 4057–4066. 10.1162/jocn_a_00078 21671745 PMC8653780

[tops12450-bib-0131] Zacks, J. M. , Speer, N. K. , Swallow, K. M. , Braver, T. S. , & Reynolds, J. R. (2007). Event perception: A mind‐brain perspective. Psychological Bulletin, 133(2), 273–293. 17338600 10.1037/0033-2909.133.2.273 PMC2852534

[tops12450-bib-0132] Zacks, J. M. , Speer, N. K. , Swallow, K. M. , & Maley, C. J. (2010). The brain’s cutting‐room floor: Segmentation of narrative cinema. Frontiers in Human Neuroscience, 4, 168. 10.3389/fnhum.2010.00168 20953234 PMC2955413

[tops12450-bib-0133] Zadbood, A. , Chen, J. , Leong, Y. C. , Norman, K. A. , & Hasson, U. (2017). How we transmit memories to other brains: Constructing shared neural representations via communication. Cerebral Cortex, 27(10), 4988–5000. 10.1093/cercor/bhx202 28922834 PMC6057550

